# Genomic diversity of prevalent *Staphylococcus epidermidis* multidrug-resistant strains isolated from a Children’s Hospital in México City in an eight-years survey

**DOI:** 10.7717/peerj.8068

**Published:** 2019-11-20

**Authors:** Roberto Cabrera-Contreras, Rosa I. Santamaría, Patricia Bustos, Irma Martínez-Flores, Enrique Meléndez-Herrada, Rubén Morelos-Ramírez, Martín Barbosa-Amezcua, Vanessa González-Covarrubias, Eugenia Silva-Herzog, Xavier Soberón, Víctor González

**Affiliations:** 1 Laboratorio de Patogenicidad Bacteriana, Departamento de Salud Pública, Facultad de Medicina, Universidad Nacional Autónoma de México, Ciudad de México, México; 2 Centro de Ciencias Genómicas, Universidad Nacional Autónoma de México, Cuernavaca, Morelos, México; 3 Instituto Nacional de Medicina Genómica, Ciudad de México, México

**Keywords:** Genomes, Pangenome, Prophages, CRISPR, Recombination, Antibiotic resistance, Insertion sequences, Clonal structure, *Staphylococcus epidermidis*, prevalence

## Abstract

*Staphylococcus epidermidis* is a human commensal and pathogen worldwide distributed. In this work, we surveyed for multi-resistant *S. epidermidis* strains in eight years at a children’s health-care unit in México City. Multidrug-resistant *S. epidermidis* were present in all years of the study, including resistance to methicillin, beta-lactams, fluoroquinolones, and macrolides. To understand the genetic basis of antibiotic resistance and its association with virulence and gene exchange, we sequenced the genomes of 17 *S. epidermidis* isolates. Whole-genome nucleotide identities between all the pairs of S. epidermidis strains were about 97% to 99%. We inferred a clonal structure and eight Multilocus Sequence Types (MLSTs) in the *S. epidermidis* sequenced collection. The profile of virulence includes genes involved in biofilm formation and phenol-soluble modulins (PSMs). Half of the *S. epidermidis* analyzed lacked the ica operon for biofilm formation. Likely, they are commensal *S. epidermidis* strains but multi-antibiotic resistant. Uneven distribution of insertion sequences, phages, and CRISPR-Cas immunity phage systems suggest frequent horizontal gene transfer. Rates of recombination between *S. epidermidis* strains were more prevalent than the mutation rate and affected the whole genome. Therefore, the multidrug resistance, independently of the pathogenic traits, might explain the persistence of specific highly adapted *S. epidermidis* clonal lineages in nosocomial settings.

## Introduction

*Staphylococcus epidermidis* (*SE*) is a typical commensal bacterium of the human skin microbiome (Byrd, Belkaid & Segre, 2018). However, some *SE* strains behave as pathogens colonizing surgery wounds, medical devices, and in some circumstances, they reach the human bloodstream causing severe bacteremia and potential mortality (Chessa, Ganau & Mazzarello, 2015; Otto, 2009). Children are especially prone to acquire methicillin-resistant *SE* strains in perinatal hospitals (Marchant et al., 2013; Villari, Sarnataro & Iacuzio, 2000). The genetic basis for pathogenicity of *SE* strains includes genes related to biofilm formation (adhesion), phenol soluble modulins (PSMs), and diverse Mobile Genetic Elements (MGEs) like phages, insertion sequences (ISs), and pathogenicity islands, that may be associated with the transfer of antibiotic and virulence traits (Bouchami, De Lencastre & Miragaia, 2016; Miragaia et al., 2009; Rolo et al., 2017) (Conlan et al., 2012; Kozitskaya et al., 2005; Meric et al., 2015). There is no clear genetic distinction between pathogenic and commensal non-pathogenic *SE* strains*,* even though nosocomial strains are enriched in virulence and antibiotic resistance genes (Conlan et al., 2012; Kozitskaya et al., 2005; Meric et al., 2018). It has been proposed that these genes are within the pool of accessory genome mobilized within and between species (Meric et al., 2015; Rolo et al., 2017). In this sense, the emergence of methicillin-resistant staphylococci has been linked to MGEs such as the Staphylococcal Chromosomal Cassette (SCC*mec*), and the Arginine Catabolic Mobile Element (ACME) that play a role in adaptation to the skin and other human surfaces (Miragaia et al., 2009; Planet et al., 2013; Rolo, Lencastre & Miragaia, 2012). Recent phylogenetic studies indicate that ACME having originated from *SE* and transferred horizontally to *S. aureus* (Barbier et al., 2011; Onishi et al., 2013; Planet et al., 2013), whereas SCC*mec* may have originated and diversified in different staphylococci species independently (Miragaia, 2018). Other factors, including, genomic rearrangements mediated by IS256 in pathogenic *SE* strains (Gu et al., 2005; Ziebuhr et al., 1999a), conjugative transfer of antibiotic resistance (Forbes & Schaberg, 1983) and the metabolic state of the staphylococci cell, may also be linked to pathogenicity (Bosi et al., 2016). Thus, pathogenicity in staphylococci may be viewed as a set of evolving genetic traits for adaptation to specific niches (Ben Zakour, Guinane & Fitzgerald, 2008; Ziebuhr et al., 1999b).

The population structure of *SE* is essentially clonal as determined by Multilocus Sequence Typing (MLST) (Miragaia et al., 2002; Miragaia et al., 2007; Thomas et al., 2007). Although, nosocomial *SE* strains show high genetic diversity among isolates from distant geographic locations (Miragaia et al., 2007) several clonal complexes are disseminated globally including the ST2, ST5, and ST23 which indeed are the most frequently found in clinical environments (Lee et al., 2018; Miragaia et al., 2007). Besides the clonal structure, recombination has been estimated to occur two times more frequently than mutation (Miragaia et al., 2007). Genomic analysis had calculated that about 40% of the core genes of *SE* had undergone recombination (Meric et al., 2015). It suggests that recombination might be a general property of *SE* for rapid evolution in clinical settings but conserving high linkage disequilibrium between alleles. In this case, recombination might act as a cohesive force, maintaining the clonal population structure but allowing clones to diverge.

Multidrug resistance is a significant concern in most health care settings worldwide because of the difficulties associated with clinical treatment and the potential of dissemination. It has been reported that the prevalent *SE* clonal lineages ST5, ST12, and ST23 often display high resistance toward most of the antibiotic classes (Martínez-Meléndez et al., 2016; Widerstrom et al., 2012). Furthermore, there is evidence of increasing of rifampicin resistance over time in clinical *SE* isolates that belong to ST2 and ST23 from the USA, Australia, and some European countries (Lee et al., 2018). Mutations in the *rpoB* gene conferring rifampicin resistance have emerged independently, suggesting the view that a limited number, well adapted multi-resistant clonal *SE* lineages, prevail in clinical settings (Lee et al., 2018; Martínez-Meléndez et al., 2016). Reports on the profiles of antibiotic multi-resistance changes over time are scarce (Lee et al., 2018). Surveillance of rifampicin resistance in a study involving 24 countries and 96 institutions suggests annual local variations for this antibiotic (Lee et al., 2018). Therefore, the correct identification of pathogenic *SE* strains and its drug resistance profile will contribute to prevent and treat these bacterial infections in the clinic.

In this work, we evaluated the prevalence of *SE* in comparison with other staphylococci species isolated in a single hospital in México City during eight years period. Then, we aimed to assess the antibiotic resistance profiles of *SE* isolates, and through genomics, to know the genome structure of a selected set of antibiotic multi-resistant *SE* strains. In this context, we provide a genome-wide measure of recombination, to shed light on the mechanisms of *SE* genetic diversification and the evolution of multidrug-resistance in local hospital settings.

## Materials & Methods

### Ethical considerations

This study was carried out following the recommendations of the ethics review committee of the Facultad de Medicina-UNAM. The *SE* strains used in this work were obtained by a donation from the microbiology collection of the Instituto Nacional de Perinatología “Isidro Espinosa de los Reyes” (INPer). A consent form was not required. Original identification keys and clinical data concerning the isolates are maintained under the control of INPer. In the present work, new strain identifiers were assigned to the INPer strains. Authors do not have access in any form to the specific clinical information of strains and patients.

### Clinical isolation of staphylococci and characterization

Staphylococci strains were recovered from primus isolates taken from patients at INPer. They were conserved frozen (−80  °C in an ILSHIN ultra-freezer). Characterization of staphylococci was carried out by streaking the cultures on tryptic soy agar plates (BD Diagnostic Systems, Germany). Plates were incubated for 18–24 h at 37 ° C to obtain isolated colonies. Individual colonies were used to inoculate tryptic soy broth and grown overnight after which genomic DNA was extracted (see below). Identification of *Staphylococcus* species was carried out using the VITEK^^®^^2 equipment (bioMérieux SA, 376 Chemin de l‘ Ome. France). Briefly, staphylococci colonies were resuspended in 0.45% saline solution and adjusted to a turbidity of 0.5 in a McFarland nephelometer. The bacterial suspension was transferred into the VITEK^^®^^2 GP ID card testing 64 biochemical properties for gram-positive bacteria, and eight specific tests for *SE* species (phosphatase and urease production, growth in 6% NaCl, resistance to novobiocin and polymyxin B, arginine hydrolysis, catalase, D-mannitol fermentation). Additional growth characteristics, biochemical (D-mannitol fermentation, coagulase, and catalase), and molecular tests (PCR detection of *coa* and *mecA*; see below) were performed at the arrival of the staphylococci collection to the Pathogenicity Laboratory at Faculty of Medicine, UNAM.

### Molecular tests

To confirm the presence/absence of the coagulase gene (*coa*), and the methicillin-coding gene (*mecA*) in *SE* and *S. aureus* strains, we did a duplex PCR according to Hookey (Hookey et al., 1999). In brief, a PCR amplification of a 177 bp fragment of the *coa* gene, with primers coa-forward 5′-AACTTGAAAT-AAAACCACAAGG-3′, and coa-reverse 5′TACCTGTACCAGCATCTCTA-3′. In the same reaction, a 458 bp fragment of the *mecA* gene was amplified using primers mecA-forward 5′-ATGGCAAAGATATTCAACTAAC-3′and mecA-reverse 5′-GAGTGCTACTCTAGCAAAGA-3′. PCR reactions were performed on a BIO-RAD C1000 Thermal Cycler (BIO-RAD, USA). The amplification conditions were as follows: 94 °C for 10 min and 30 cycles at 94 °C for 30 s, 56.2 °C for 30 s and 72 °C for 30 s and a final amplification at 72 °C for 10 min; PCR products were separated by electrophoresis in a 1% agarose gel stained with ethidium bromide and visualized with UV light.

### Antibiotic resistance

The antibiotypes for 17 antibiotics were determined using automatic VITEK^®^2 equipment with panel card AST-GP577 (VITEK^®^2 Biomerieux, Francia). Resistance and susceptibility patterns to 17 antimicrobials were determined according to the M100S Performance Standards for Antimicrobial Susceptibility Testing (Clinical and Laboratory Standards Institute C, 2019). The following antibiotics were tested using the respective reference MIC: Amoxicillin/Clavulanic Acid: ≤4/2 µg/mL, Ampicillin: >8 µg/mL, Cefazolin: ≤2 µg/mL, Ciprofloxacin: ≥4 µg/mL, Clindamycin: >4 µg/mL, Erythromycin: >8 µg/mL, Gentamicin: >16 µg/mL, Imipenem: 8 µg/mL, Levofloxacin: ≥4 µg/mL, Linezolid: ≥8 µg/mL, Oxacillin: ≥0.5 µg/mL, Penicillin: ≥0.25 µg/mL, Rifampicin: ≥4 µg/mL, Synercid: > µg/mL, Tetracycline: ≥16 µg/mL, Trimethoprim/Sulfamethoxazole: >4/76 µg/mL, and Vancomycin: ≥32 µg/mL.

Methicillin resistance was evaluated by the disc diffusion method for cefoxitin on Mueller-Hinton agar plates (OXOID Ltd, Basingstoke, Hampshire, England), and with the plate method in Mueller-Hinton agar plates supplemented with 6 µg/ml of oxacillin, according to the standards from the Center for Disease Control and Prevention, USA (Centers for Disease Control and Prevention C, 2019)

### Genome sequencing

Genomic DNA was isolated by using the Lysostaphin-Lysozyme method of Hookey (Hookey et al., 1999), only modifying the lysis step. Briefly, bacterial cells were resuspended in 250 µl of lysis solution (lysozyme 50 mg/ml; lysostaphyn 1 mg/ml in 25 nM Tris-HCl- 10 mM EDTA; all reagents from SIGMA-ALDRICH, St. Louis MO USA), and incubated at 37  °C by 90 min. The sample was then warmed up at 95  °C for 10 min and centrifuged at 8,000× g, after which the sample was processed as indicated in the method.

DNA integrity and purity were assessed by agarose gel electrophoresis and spectrophotometry (NanoDrop 2000; ThermoFisher, Waltham, MA, USA); final concentrations were assessed using the Qubit high sensitivity dsDNA assay (ThermoFisher). Libraries were generated with Nextera XT DNA library preparation kit (Illumina, San Diego, CA, USA), according to the manufacturer’s protocol, using 1 ng of DNA. Libraries quality were evaluated on an Agilent 4200 TapeStation system (Santa Clara, CA, USA) and sequenced using a NextSeq 500/550 mid output kit (2 × 150 bp) (Illumina), at an estimated depth of coverage of 100x.

### Genome assembly and annotation

Assemblies were made with Spades 3.6.0 in genomes sequenced in high coverage (50x to 100x). Genome annotations were obtained from the PATRIC server (Wattam et al., 2018) (https://www.patricbrc.org/), through the automated bioinformatic method RAST (Rapid Annotation Subsystem Technologies) (Antonopoulos et al., 2019). Gene annotation of antibiotic resistance and virulence-related genes were obtained from the section of Special Genes of PATRIC. Annotations for virulome were supported with other sources of information such as PATRIC VF, VFDB, and Victors. For the prediction of antibiotic resistance genes, the PATRIC server was used to search specialty genes using the databases CARD (Comprehensive Antibiotic Resistance Database) and NDARO (National Database of Antibiotic Resistant Organism) (Davis et al., 2016).

### Genome comparisons and pangenome modeling

Average Nucleotide Identity by MUMmer (ANIm) and Genomic Coverage (G _cov_) were calculated with the JSpecies program, with MUMmer used as a pairwise comparison tool for pairs of *SE* genomes (Richter et al., 2016). The pangenome was modeled using the GET_HOMOLOGUES package and its default values (Contreras-Moreira & Vinuesa, 2013). Briefly, groups of orthologues proteins were computed using the Ortho-MCL integrated into GET_HOMOLOGUES. Accessory genes (unique genes and genes present in at least in two genomes but not in all genomes), were obtained from the cloud, shell, and soft-core pangenome components according to GET_HOMOLOGUES (Contreras-Moreira & Vinuesa, 2013). The distribution of accessory genes in *SE* genomes was illustrated with the heatmap.2 function of the R’s ggplot2 package.

### Phylogeny

A consensus core genome calculated from the pangenome model of 29 *SE* genomes was obtained with GET_HOMOLOGUES (Contreras-Moreira & Vinuesa, 2013). The resulting 1575 core protein clusters were subject to multiple alignments with MUSCLE (Edgar, 2004), gaps removed with TrimAl v2.1, and were concatenated using homemade Perl scripts (Capella-Gutiérrez & Gavaldón, 2013). Phylogenetic trees were constructed by the maximum likelihood (ML) method based on the substitution matrix of WAG (Whelan and Goldman model), with 1000 bootstrap replicates, using RaxML program (Stamatakis, 2015). To draw and edit the phylogenetic trees, we used the iTool program (Letunic & Bork, 2019).

### Recombination

The inference of recombination was performed with ClonalFrameML (Didelot & Wilson, 2015). First, we ran GET-HOMOLOGUES to obtain the common protein clusters encoded by the genomes of each set of *SE* strains (Contreras-Moreira & Vinuesa, 2013). Second, they were converted to nucleotide sequences and concatenated using homemade Perl scripts. Multiple alignments were made as described above for phylogeny construction. Third, a RAxML (Stamatakis, 2015) tree was done to obtain the Newick format and the transition/transversion parameter *κ* for running ClonalFrameML under default parameters (Didelot & Wilson, 2015). Z-score statistics were obtained for all the sets of *SE* genomes and p-values using the web application Z Score Calculator (https://www.socscistatistics.com/tests/ztest/zscorecalculator.aspx). BoxPlots were performed with the R ggplot2 system.

### Mobile elements identification

Prophages were identified with PHAST (Arndt et al., 2019). Only predictions ranked as “intact prophages” were considered for analysis. IS, CRISPR-Cas elements and spacer sequences were obtained from PATRIC annotations (Antonopoulos et al., 2019). Then, IS were classified into families by BLASTx comparison with the ISfinder (Siguier et al., 2006). The phage identity of the spacers within the CRISPR-Cas elements was determined by BLASTn comparisons with the NCBI virus database. Only identical matches with a phage sequence in the database were recorded (Páez-Espino et al., 2016).

### Identification and classification of SCC *mec* and ACME proteins

All the predicted proteins that compose the 12 types of SCC*mec* and the six ACME types were downloaded from GenBank (Table S1) and compiled in a local protein database (SE-17db). Then, all encoded proteins of the 17 *SE* genomes were used as a query in BlastP similarity searches with the SE-17db. Best-blast hits with a minimum similarity of 80% and 60% of coverture over the length of the smaller protein were taken as evidence of homology with the respective SCC*mec* or ACME type. To classify the SCC*mec* types, we used the phylogenies of the recombinases *ccr* A, B, and C, performed according to the method described above. ACME types were classified by the presence/absence of the genes of the operons *arg*, *opp*, *ars*, and *kpd* according to a reference table (Table S2).

### GenBank accession numbers

*S. epidermidis* of the INPer collection used in this work were uploaded in GenBank with the following Biosample identifiers: SAMN11086744, SAMN11086745, SAMN11086746, SAMN11086747, SAMN11086748, SAMN11086749, SAMN11086750, SAMN11086751, SAMN11086752, SAMN11086753, SAMN11086754, SAMN11086755, SAMN11086756, SAMN11086757, SAMN11086758, SAMN11086759, SAMN11086760. The accession numbers for the genomes of reference *S. epidermidis* strains are listed in Table S3.

## Results

### Survey of antibiotic multidrug-resistant intrahospital staphylococci in eight years period

We studied the incidence of staphylococci species and strains multi-resistant to antibiotics in the INPer children hospital from 2006 to 2013. A total of 822 staphylococci strains were recovered from distinct infection sites of newborns and adults. *Staphylococcus* species were identified using the VITEK^^®^^2 system and standard clinical methods, including tests for biofilm formation, coagulase reaction, presence of *coa* and *mecA* gene, and resistance to 17 antibiotics (see methods). The analysis showed that *SE* strains were the most abundant (573 strains), followed by *S. aureus* (146 strains), and other coagulase-negative *Staphylococcus* (CoNS) species in low proportion (81 strains) (Fig. S1). The *SE* group was composed mainly of strains recovered from blood samples and catheters (Fig. S1).

**Figure 1:**
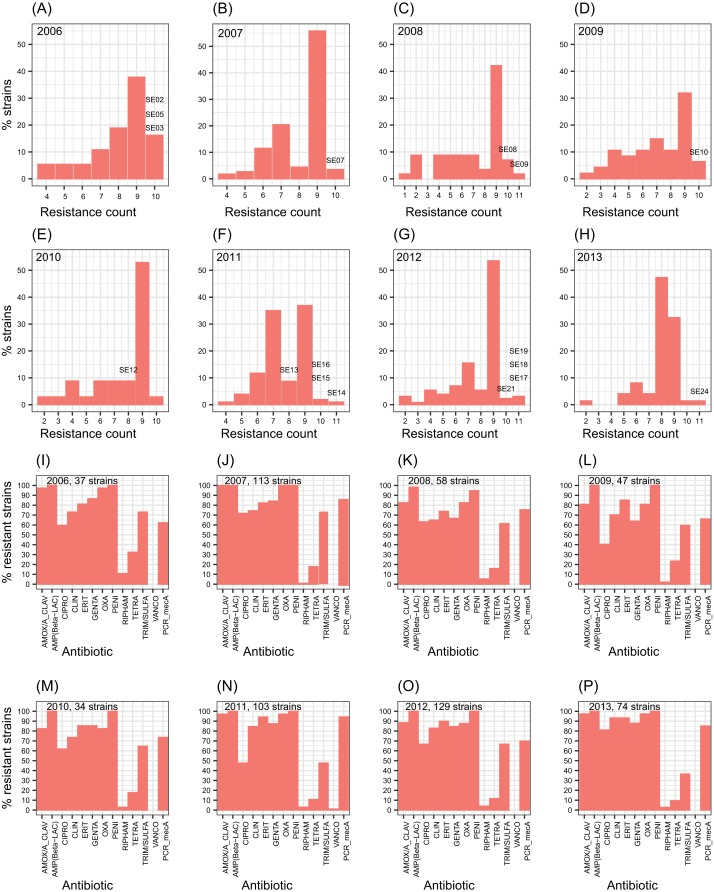
Profiles of *S. epidermidis* antibiotic multi-resistance in an eight years period. (A–H) percentage of *SE* strains respect the count of antibiotic resistances by year. (I–O) number of strains resistant to each one of twelve antibiotics by year. Percentage of *mecA* strains are shown in the last columns of each plot. The *SE* strains selected for genome analysis are indicated over the bars.

The number of antibiotic multi-resistant *SE* strains per year showed similar profiles through the studied period (Figs. 1A–1H). Methicillin resistance was found in about 80% of the *SE* isolates. *SE* strains multi-resistant to nine antibiotics were the most frequently found throughout the eight years. In contrast, *S. aureus* strains showed resistance to about four antibiotics as the most common profile (Fig. S2). Similarly, the resistance to each antibiotic remained without change, during the eight-year study (Figs. 1I–1P). These results suggest the persistence of a multidrug-resistant clone or few clones of *SE* at the hospital during the eight years*,* or frequent horizontal exchange of genes encoding antibiotic resistance between *SE* clones. To study these alternatives, we choose one or more *SE* strains per year to totalize 17 clinical *SE* coagulase-negative (CoNS) strains (Fig. 1). These particular strains came from nosocomial infections of 14 newborns and three adults, isolated from three different infection sites: blood (8 strains), catheters (7 strains), cerebrospinal fluid (1 strain), and soft tissue (1 strain) (Table 1).

**Table 1:** General features of the genomes of *S. epidermidis* strains.

									***ccr*recombinases in*SCCmec*type**				
**Strain**^a^	**Origin**^b^	**ST**^c^	**ST profile**^d^	***ica***	**CRISPR-Cas**	**Prophages**	**IS256**^e^	***mecA***	***ccrA***		***ccrB***		***ccrCf***
S02	N-Catheter	23	7,1,2,1,3,3,1	+	1	–	1 (1)	+	IV	–	IV	–	IV
S03	N-Catheter	89	1,1,2,1,2,1,1	–	0	+	1 (1)	+	IV	–	IV	–	–
S05	N-Catheter	23	7,1,2,1,3,3,1	+	1	–	1 (1)	+	IV	–	IV	–	IV
S07	A-STA	59	2,1,1,1,2,1,1	–	0	+	–	+	IV	VIII	IV	VIII	–
S08	N-Blood	23	7,1,2,1,3,3,1	+	1	–	1 (1)	+	IV	–	IV	–	–
S09	A-Blood	59	2,1,1,1,2,1,1	–	1	–	–	+	IV	VIII	IV	VIII	–
S10	A-Blood	U-ST	65,48,5,5,8,5,11	–	0	–	–	–	–	–	–	–	–
S12	N-Blood	5	1,1,1,2,2,1,1	–	3	+	1 (1)	+	IV	–	IV	–	IV
S13	N-Blood	81	2,17,1,1,2,1,1	–	1	–	1(0)	+	IV	VIII	IV	VIII	NA
S14	N-Blood	2	7,1,2,2,4,1,1	+	0	+	2 (1)	+	IV	–	IV	–	–
S15	N-Catheter	5	1,1,1,2,2,1,1	–	0	+	1 (1)	+	IV	–	IV	–	NA
S16	N-Blood	2	7,1,2,2,4,1,1	+	0	+	1 (1)	–	–	–	–	–	–
S17	N-Catheter	2	7,1,2,2,4,1,1	+	0	–	2 (1)	+	IV	–	IV	–	–
S18	N-Blood	2	7,1,2,2,4,1,1	+	0	+	2 (1)	+	IV	–	IV	–	–
S19	N-Catheter	2	7,1,2,2,4,1,1	+	0	–	2 (1)	+	IV	–	IV	–	–
S21	N-Catheter	35	2,1,2,2,4,1,1	+	1	–	–	+	–	–	–	–	–
S24	N-CSF	5	1,1,1,2,2,1,1	–	3	+	–	+	IV	–	IV	–	–

**Notes.**

a S05 lack of *icaC*.

b N prefix indicates strains isolated from neonates; A, isolates from adults; CSF, cerebrospinal fluid; STA, soft tissue aspirate.

c U-ST, unassigned ST.

d ST profile numbers correspond to alleles of ArcC, AroE, Gtr, MutS, TpiA, and YqiL, according to Thomas et al. (2007).

e Parentheses indicate the number of complete IS256.

f NA, indicates the presence of a *ccrC* homologue but not assigned to any SSC*mec* type.

### Broad gene catalog from draft genomes

We obtained the whole genome sequence of 17 nosocomial *SE* strains (see ‘Material & Methods’). After testing different parameters with the assembler programs Velvet and Spades (Bankevich et al., 2012; Zerbino, 2010), we got draft genomes assemblies consisting of about 92 up to 432 contigs with a 60–70×average sequence coverture per genome (Table S4). To assert that the 17 assemblies represent a substantial part of the *SE* genomes, we compared the total genome length, and the number and length of the predicted ORFs, with 11 complete genomes of *SE* downloaded from GenBank (Table S5). There were no differences between the genome length of the *SE* INPer genomes and the complete genomes from the GenBank (unpaired *t*-test = 2.33; *p*-value = 0.022), in ORFs number (unpaired *t*-test = −1.68; *p*-value = 0.117), or ORFs length (unpaired *t*-test = 2.60; *p*-value = 0.014) (Table S5). Therefore, we can conclude that the *SE* INPer genome sequences obtained here provide a broad catalog of genes per genome useful for comparative genomics.

### Genomic, pangenomic, and phylogenetic relationships among *SE* isolates

To define the genomic similarity between the 17 clinical *SE* strains, we performed whole-genome pairwise nucleotide identity estimates (Average Nucleotide Identity by Mummer, ANIm) (Richter et al., 2016). The 17 *SE* showed high genomic ANIm values of about 99%, covering more than 90% of the genome length (Fig. S3). One exception is strain S10, which showed ANIm values of about 97% concerning the rest of the strains.

Besides the genomic identity between *SE* isolates, we wanted to investigate the extent of their genetic variability by performing a pangenome model. To this end, the *SE* collection was complemented by the inclusion of 12 complete genomes of *SE* strains downloaded from GenBank (Table S3, NCBI reference complete genomes). The model obtained using the GET_HOMOLOGUES software package (Contreras-Moreira & Vinuesa, 2013), indicated an open pangenome (Fig. S4). The core genome component for the 29 *SE* strains was predicted to consist of about 1,575 gene clusters, whereas the sum of genes unevenly distributed in the 29 *SE* genomes (accessory component) contains 4,360 gene clusters.

**Figure 2:**
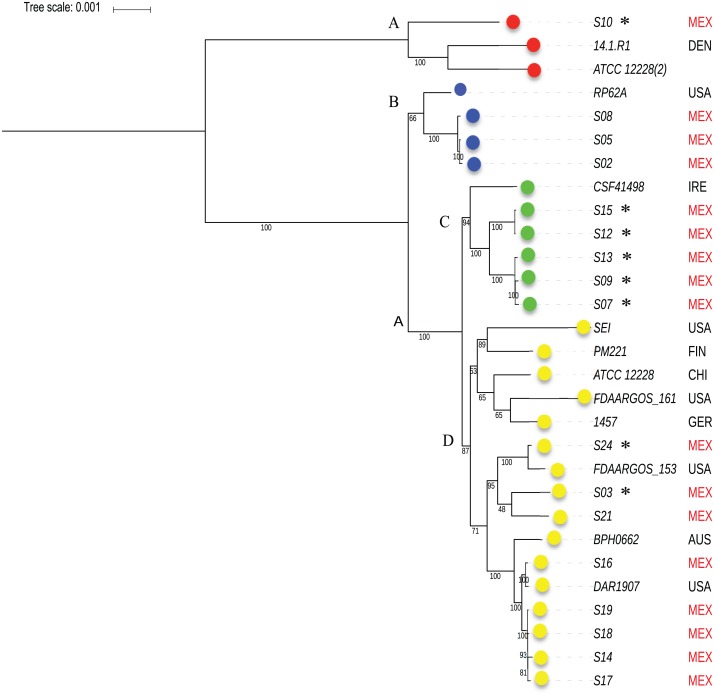
Phylogenetic relationships of *S. epidermidis* from the Children’s Hospital (INPer) and *S. epidermidis* selected from GenBank. Phylogenetic relationships of *S. epidermidis* from the Children‘s Hospital (INPer) and *S. epidermidis* selected from GenBank. The ML tree consists of four main clades defined by the longest branches (A to D). They are indicated with color dots: red, clade A; blue, clade B; green, clade C; yellow, clade D. The tree was constructed with 1,575 common core proteins using RaxML program as described in methods. The results of bootstrap performed with 1,000 replicates are indicated in the branches. Acronyms specify the isolation site of the *SE* strains: MEX, México; USA, United States of America; FIN, Finland; GER, Germany; DEN, Denmark; IRE, Ireland; CHI, China; AUS, Australia. *SE* INPer strains are denoted in red. Asterisks indicate *ica*^−^** strains.

To know the phylogenetic relationship of the *SE* from INPer in the context of reference *SE* strains, we did an un-rooted ML phylogenetic tree using the predicted 1,575 concatenated core proteins (Fig. 2). There were three clades separated by the largest branches in the tree that comprise most of the *SE* local strains, and one or more *SE* strains isolated worldwide (Fig. 2, clades B, C, D). The clade marked as D in the tree consisted of two groups, one of which includes only reference *SE* strains, whereas the other had most of the *SE* strains of the collections studied. Strain S10, the most different strain by ANIm in the *SE* collection, was grouped in the clade A with the commensal strains *SE* ATCC12228 (2) and *SE* 14.1.R1 isolated from USA and Denmark.

**Figure 3:**
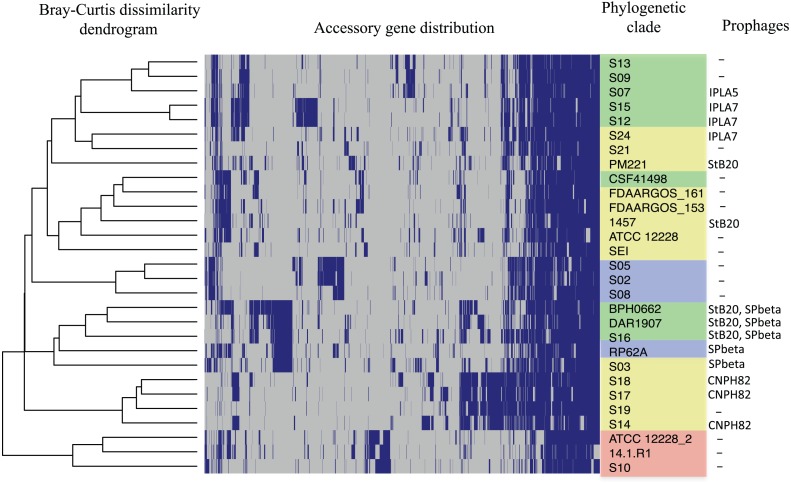
Distribution of accessory genes in the *S. epidermidis* genomes. The heat-map profile was performed with the ggplot2 function in R using the Bray-Curtiss dissimilarity matrix. The Bray–Curtiss dendrogram is indicated at the left. The heat-map at the middle indicates gene presence (blue color); empty cells represent the absence of genes. *S. epidermidis* strains are shown at the left with the same colors of the clades in the core proteins phylogeny (Fig. 1): red, clade A; blue, clade B; green, clade C; yellow, clade D. In the last column. the presence/absence of genomic regions similar to known prophages (Table S5) is indicated.

The distribution of accessory genes in individual *SE* strains coincided with the phylogenetic clades. The *SE* strains grouped in three phylogenetic clades are closely related by their similitude in the gene presence/absence profile (Fig. 3). Despite the high identity of the *SE* strains, there is still considerable individual variation that may account for adaptations to local milieu.

### Clonal structure

To investigate to which clonal ST complex the *SE* strains belong, we looked for the seven proteins of the *S. epidermidis* MLST scheme, and compared them with their respective alleles in the *Staphylococcus epidermidis* MLST database (https://pubmlst.org/sepidermidis/; Table 1; see methods) (Feil et al., 2004; Thomas et al., 2007). The analysis showed a total of 8 different STs; seven of them already recorded in the database. The S10 strain had an unassigned ST in the database and only differed by a single amino acid substitution in the YqiL protein (L370C). The ST2, ST5, and ST23 are worldwide distributed and are the most represented in our sample (Lee et al., 2018; Miragaia et al., 2007). In agreement with global data, ST35, ST59, ST81, ST89 are less frequently represented. The clonal relationships among STs determined by eBURST, indicate that founder clones are ST2 and ST5, whereas the other four STs (ST 59, 81, 89 and 23), are peripheral clones mostly related to each than to the primary founder clones (Fig. S5).

### Virulence genes

Several known virulence genes of *SE* are shared by commensal and pathogenic strains (Otto, 2009). Among them, we found the cluster *icaADBCR* (biofilm formation) in nine out of the 17 *SE* genomes analyzed, except for *icaC,* absent in strain S05 (Table 1). The phenol-soluble modulins (PSMs), involved in inflammatory response and lysis of leukocytes were present in the genomes of all *SE* strains (Peschel & Otto, 2013). The novel ESAT 6 (*esaAB*, *essABC, and esxAB* genes*)* secretion system implicated in immune system evasion and neutrophil elimination was only present in S10 strain (Burts et al., 2005; Dai et al., 2017; Wang et al., 2016).

Frequently, *SE* isolates contain the genomic island ACME, composed by arginine deaminase catabolic (*arg*) and an oligopeptide permease operons (*opp*) (Miragaia et al., 2009). ACME has been associated with successful adaptation to the skin and mucosal surfaces outcompeting other related bacteria (Planet et al., 2013). The diversity of ACME has been organized in six distinct types according to their gene composition (Table S2 and references therein). We looked for the presence of ACME genes in the 17 *SE* genomes of INPer collection. By means of BlastP comparisons we identified genes belonging to the ACME Type I (*arg*^+^ *opp*^+^) in genomes of *SE* strains S03, S17, and S21, and the ACME Type V that contain the *arg*^+^ *opp*^+,^ and *ars*^+^ (arsenate resistance operon), and *kpd+*, a potassium transporting operon in S24 (Centers for Disease Control and Prevention C, 2019; O’Connor, McManus & Coleman, 2018) (Table S2). Even though some other *SE* strains present genes of the *arg* and *ars* operon, they lacked the genes of the *opp* operon; therefore, they were not assigned to ACME known types.

### Antibiotic resistance genotype and phenotype

The *SE* strains were tested for their susceptibility to methicillin and other *β*-lactams antibiotics as described in methods. All the 17 *SE* strains have the *β*-lactamase gene (*blaZ*) and their regulators (*blaR* and *I*), which are probably responsible for the broad resistance spectrum to penicillin, carbapenems, and cephalosporins determined by the VITEK^^®^^2 system (Table 2). Methicillin resistance was evaluated by the disc diffusion method for cefoxitin and corroborated by the presence of *mecA* in the genomes (Table 1 and Table 2). 12 out of 17 *SE* strains showed agreement between the cefoxitin resistance phenotype and the *mecA* genotype. Although strains S03, S05, S14, S15, and S21, have the *mecA* gene in the genome, they were scored as susceptible for cefoxitin (disc diffusion ≥ 25 millimeters). To support that the 15 *mecA* positive strains are Methicillin-Resistance *SE* (MRSE) strains, we evaluated their resistance to oxacillin. Commonly, the Methicillin-Resistance *S*. *aureus* (MRSA) strains are evaluated by the resistance to oxacillin (Centers for Disease Control and Prevention C, 2019). In the *SE* collection, only the S07 strain was susceptible to oxacillin (6 µg/ml) in plate assays, despite the detection of *mecA* in the genome. Altogether, these results indicate that most of the *SE* strains (15) studied here are MRSE, identified by the presence of the *mecA* gene; any phenotypic inconsistencies could be due to lack of expression of the *mecA*, heteroresistance, or the limitation of the current antibiotic susceptibility testing methods (Band et al., 2019; Harrison et al., 2019; Nicoloff et al., 2019).

**Table 2:** Antibiotic resistance phenotypes and the genotype profile in *S. epidermidis* strains. Resistant strains are indicated by gray cells (+). Susceptible strains are in blue cells (−). Strains not tested are indicated as empty cells. Parentheses in the Genotype column indicates the number of strains that present the most probable gene (s) involved in antibiotic resistance. In *gyrA* and *rpoB* the corresponding amino acid substitutions in the protein are indicated. The S10 and S16 strains are denoted as *mecA*- by an asterisk. Cefoxitin and Oxacillin antibiotics used for phenotypic characterization of methicillin resistance are marked **by a rectangle in the first column**. Cefoxitin was evaluated by the disc-diffusion method, considering growth inhibition zones of ≤25 mm as susceptible, and ≥24 mm, resistant (Centers for Disease Control and Prevention C, 2019).

		***S. epidermidis*strains**																	**Genotype**
**Antibiotic**	**Class**	**S02**	**S03**	**S05**	**S07**	**S08**	**S09**	S10*	**S12**	**S13**	**S14**	**S15**	S16*	**S17**	**S18**	**S19**	**S21**	**S24**	Genes probably involved
Ampicillin	Penicillin	+	+	+	+	+	+	+	+	+	+	+	+	+	+	+	+	+	*blaZ* (17), *mecA* (15)
Penicillin	Penicillin	+	+	+	+	+	+	+	+	+	+	+	+	+	+	+	+	+	*blaZ* (17), *mecA* (15)
Amoxicillin	Penicillin	+	+	+	+	+	+	+	+	+	+	+	+	+	+	+	+	+	*blaZ* (17), *mecA* (15)
**Oxacillin**	**Penicillin**	+	+	+	–	+	+	–	+	+	+	+	–	+	+	+	+	+	*blaZ* (17), *mecA* (15)
**Cefoxitin**	**Penicillin**	–	–	+	–	+	+	–	+	+	–	–	–	+	+	+	–	+	*blaZ* (17), *mecA* (15)
Imipenem	Carbapenem	+	+	+	+	+	+	+	+	+	+	+	+						*blaZ* (17), *mecA* (15)
Cefazolin	Cefalosphorin	+	+	+	+	+	+	+	+	+	+	+	+						*blaZ* (17), *mecA* (15)
Ciprofloxacin	Quinolone	+	–	+	–	+	+	+	–	–	+	+	+	+	+	+	+	+	*gyrA* S84F (10)
Levofloxacin	Quinolone	+	–	+		+	+	+	–	–	+	+	+	+	+	+	+	+	*gyrA* S84F (10)
Erythromycin	Macrolide	+	+	+	+	+	+	+	+	+	+	+	+	+	+	+	+	+	*mphB* (3), *ermC* (11)
Clindamycin	Lyncosamide	+	+	+	+	+	+	+	+	–	+	+	+	+	+	+	+	+	*linA* (1), *ermC* (11)
Tetracycline	Tetracicline	–	+	–	+	–	+	–	–	+	+	–	–	+	+	+	–	–	*tetL* (4)
Gentamycin	Aminoglycoside	+	+	+	+	+	+	+	+	+	+	+	+	+	+	+	+	+	*aac/aph* (15), *aph3* (2), *ant9* (3), *aadD* (11)
Trimethoprim/SMX	Sulfonamina	+	+	+	+	+	+	+	–	–	+	+	+	+	+	+	+	+	*sul3* (17)
Chloramphenicol	Phenicol	+	–	+		+	+	+	+	–	+	+	+						*catB* (6)
Rifampicin	Miscellaneus	+	+	+	+	+	+	+	+	+	+	+	–	+	+	+	+	+	*rpoB* D471E, I527M (3); I527M (4)
Vancomycin	Glycopeptide	–	–	–	–	–	–	–	–	–	–	–	+	–	–	–	–	–	*vanRI* (17)

The gene *mecA* encodes for a penicillin-binding protein (PBP) carried in a mobile element known as Staphylococcal Chromosomal Cassette or SCC*mec* (International Working Group on the Classification of Staphylococcal Cassette Chromosome E, 2009)*.* By localizing the recombinases *ccrA*, *B*, and *C*, as well the *mecA* genes in the contigs of the respective genomes, and then constructing phylogenies including known SCC*mec* recombinase genes, we classified the SCC*mec* types of the *SE* INPer strains (Table 1; Fig. S6). The analysis demonstrated the presence of the community-acquired SCC*mec* type IV cassette in 13 out of 14 methicillin-resistant strains. The *SE* INPer strains S10 and S16 lack the SCC*mec* cassette, and no *mecA* gene was detected. Although the S21 strain has a *mecA* gene, we were unable to find other gene elements to show the presence of a *mec* cassette. Moreover, strains S07, S09, S13 strains contain an additional SCC*mec* type VIII cassette in tandem with the SCC*mec* IV cassette. The S07 strain carries a contig of 36 535 bps of the SCC*mec* type IV and VIII, suggesting the probable structure of the recombined cassette (Fig. S7).

The genomic analysis revealed that some *SE* strains studied here included genes for resistance to fluoroquinolones, macrolides, sulfonamides, aminoglycosides, tetracycline, and other antibiotics not used as the first choice in clinical therapy (Table 2). The results given in Table 2 corroborate that in most of the cases, the probable gene responsible for the resistance is present in the genomes. Besides, non-synonymous mutations in the antibiotic target proteins GyrA and RpoB were identified in some *SE* strains resistant to quinolones and rifampicin. Despite other *SE* strains lack these mutations, they still were resistant to these antibiotics. Then, other mutations in the antibiotic target protein or other genetic mechanisms not yet known would be responsible for these resistances.

### Mobile genetic elements

The genomic variability observed in the *SE* strains suggests active processes of recombination and gene exchange. To study this concern, we first look for prophages and CRISPR-Cas related systems in the genomes. Prophage footprints were found in 10 out of 17 genomes of the *SE* strains. The most significant prophage hits detected by the PHAST program, were for genomic regions spanning about 28 to 95 kb that include an attachment site, a signature of lysogenic phages (Arndt et al., 2019) (Table S6). In this analysis, prophages were found integrated into the genomes of some *SE* strains, such as CNPH82 found in the *SE* strains S14, S17, and S18 (Daniel, Bonnen & Fischetti, 2007). Some other prophages such as StB20 and SpBeta were present in S03 and S16 strains respectively and, the prophages IPLA5 in strain S07 and IPLA7 in S12 and S15 strains (Gutiérrez et al., 2012). In the remaining *SE* strains, prophages sequences were not detected.

*SE* strains have also acquired defense mechanisms against phage infection. The search for CRISPR-Cas immune systems identified nine out of the 17 *SE* INPer strains carrying a CRISPR-Cas Type III system. The system III is composed by the Cas1 and Cas2, responsible for spacer processing and insertion; the ribonuclease Cas6, and the cascade proteins Csm1 to Csm6, involved in the processing of the target transcript (Table S7). The CRISPR-Cas Type III system has been already reported to confer immunity to phages as well as to conjugative plasmids in *SE* (Marraffini, 2015; Marraffini & Sontheimer, 2008). Although the enzymatic organization of the CRISPR-Cas systems is remarkably conserved in *SE*, there are variations in the array of repeats and spacers in the CRISPR loci. Three distinct types of identical repeated units of 30 or 36 nucleotides, associated with specific sequence spacers have been described (Marraffini, 2015). Three spacers that correspond to the CRISPR loci found in the strains S02, S05, and S24, match precisely with a sequence in the *Staphylococcus* phage PH15 genome for the first two strains and the *Staphylococcus* phage 6ec genome for the last (Aswani et al., 2014; Daniel, Bonnen & Fischetti, 2007).

*SE* strains harbor many ISs, belonging to different families (Table S8). The presence of IS256 has been found within pathogenic *SE,* associated with biofilm formation and virulence (Kozitskaya et al., 2004; Murugesan et al., 2018). Among the *SE* strains of our collection, the isolates having the IS256 also contain the *ica* operon, confirming previous observations. Exceptionally, only the strain S21 has the *ica* genes but lacks IS256.

### Recombination

The above results suggest that high frequency of Horizontal Gene Transfer (HGT) and recombination might promote diversification of local *SE* populations. To evaluate this, we measured the ratio of recombination to mutation (r/m), using the ClonalFrameML program (Didelot & Wilson, 2015) in the 17 INPer *SE* genomes and 16 combined sets of *SE* genomes downloaded from GenBank (Fig. 2; Table S3). A median average r/m rate of about 6.9 was calculated when the 17 *SE* were tested, suggesting that nucleotide substitutions by recombination are more frequent than random point mutations (Vos & Didelot, 2009). Every COG class shows r/m values equal or higher than the estimate for the complete set of 17 *SE* genomes. Indeed, the r/m values on virulence or antibiotic resistance gene class result similar to the other COGs involved in housekeeping functions.

**Figure 4:**
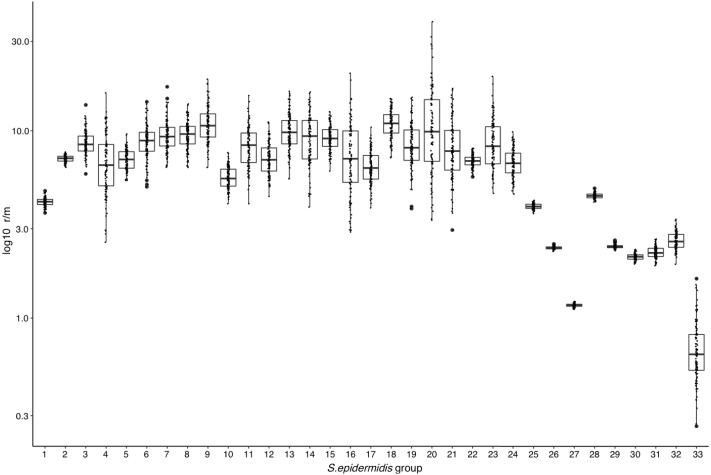
Rates of recombination/mutation for *S. epidermidis* INPer strains compared with sets of *S. epidermidis* from GenBank. 1. Sixteen strains out 17 *S. epidermidis* of the INPer collection; 2. Seventeen *S. epidermidis* strains of the INPer collection. 3–22, r/m rates for the genes encoding proteins classified in COGs in the 17 *SE* INPer: 3. COG C (energy production). 4. COG D (cell division). 5. COG E (amino acid transport and metabolism); 6. COG F (nucleotide transport and metabolism). 7. COG G (carbohydrate transport and metabolism). 8. COG H (coenzyme transport and metabolism). 9. COG I (lipid transport and metabolism). 10. COG J (translation, ribosomal structure, and biogenesis). 11. COG K (transcription). 12. COG L (replication, recombination, and repair). 13. COG M (cell wall, membrane, and envelope biogenesis). 14. COG O (post-translational modification, protein turnover, and chaperones). 15. COG P (inorganic ion transport and metabolism). 16. COG Q (secondary metabolites biosynthesis, transport, and catabolism). 17. COG R (general function predicted). 18. COG S (function unknown). 19. COG T (signal transduction mechanisms). 20. COG U (intracellular trafficking, secretion, and vesicular transport). 21. COG V (defense mechanism). 22. Unassigned COGs. 23. Antibiotic resistance genes predicted in PATRIC server for the *SE* INPer strains. 24. Virulence genes predicted in PATRIC server for the *S. epidermidis* INPer strains. 25. Reference set of 12 complete genomes of *S. epidermidis* strains used through this work. 26–33, subsets of draft *SE* strains from GenBank: 26. Ctr1 (*n* = 36). 27. Ctr2 (*n* = 26). 28. Ctr3 (*n* = 22). 29. Ctr4 (*n* = 35). 30. Ctr-5 (*n* = 36). 31. Ctr6 (*n* = 26). 32. Ctr7 (*n* = 36). 33. Ctr8 (*n* = 36). Descriptions of the *SE* strains included in the control sets and their GenBank accession numbers are shown in Table S3.

To determine whether or not the recombination estimates were affected by the sample composition of *SE* strains, we design several control tests, with distinct groups of genomes. First, we discarded the most divergent S10 strain of the *SE* collection and ran the ClonalFrameML test only with the 16 *SE* most related *SE* strains of the collection. As shown in Fig. 4, the r/m rate for this set reduced to a median of four, and this value is not significantly different respect to the r/m of *SE* of the complete genome of *SE* strains obtained from the GenBank (z-score = 1.4, *P*-value 0.135) (Fig. 4, boxplot 25). Second, we computed the r/m rate in eight different sets randomly selected among 260 complete and draft genomes of *SE* strains from the GenBank (Fig. 4, 26–33; Table S3). Some sets (Ctr2 and 8) display the lowest r/m values, whereas the rest control sets have r/m upper than two up to four. These results indicate that the strains sample composition influence the recombination estimates.

**Figure 5:**
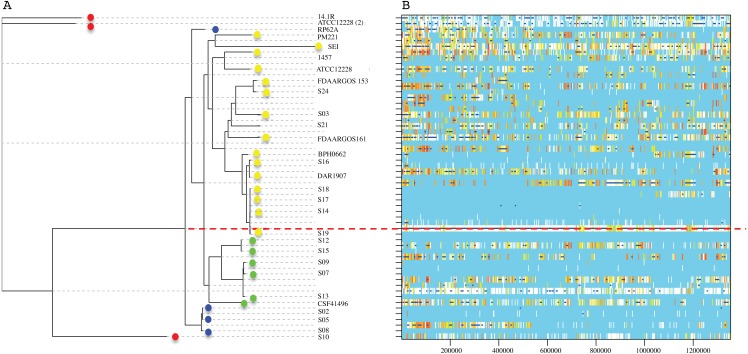
Genome-wide recombination between *S. epidermidis* strains. ClonalFrameML program detected several events of recombination along 1,354,455 concatenated genomic regions of 29 *S. epidermidis* genomes (17 INPer genomes and 12 ** GenBank genomes). (A) RaxML nucleotide tree is shown at the left of the scheme. Color dots indicate the corresponding clades in the protein phylogeny (Fig. 1). (B) Blue bars indicate recombination events along the concatenated genome segments. White bars indicate non-homoplasic nucleotide substitutions; yellow to red bars are probable homoplasic nucleotide substitutions (Didelot & Wilson, 2015). Red dots line indicate an ancestral event of recombination.

The RaxML nucleotide phylogenetic tree used as a reference to estimate recombination looks similar to the core protein phylogeny presented in Fig. 2; *SE* strains within clades maintain cohesive relationships (Fig. 5). However, multiple recombination events were detected in the ancestral nodes leading to the *SE* strains. The most prominent branch (red dot line in Fig. 5) divided the *SE* strains into two large clades, one including the clade D and the other constituted by clade B and C. Within different divergent lineages recombination introduces much more nucleotide variants than mutation as presented before (r/m). These results indicate that local hospital settings *SE* strains may contain enough genomic diversity despite their close relationship with the main clonal ST complexes of worldwide distribution.

## Discussion

*SE* is among the most common bacterial isolates found in the human skin microbiome (Byrd, Belkaid & Segre, 2018). It is also frequently recovered from bacteremia and sepsis samples in neonatal care clinic units, being its most probable etiological agent (Byrd, Belkaid & Segre, 2018; Otto, 2009). In clinical practice, it is difficult to assess if *SE* clones are the causal agents of the disease, are accidental, or opportunistic pathogens (Miragaia et al., 2008; Otto, 2017). In this work, we conducted a survey at the Instituto Nacional de Perinatología “INPer” in México City, over a period spanning eight years, to register changes in the antibiotic profile of staphylococci species and strains. We focus on *SE* because it was the bacteria most frequently found among the staphylococci isolates and showed a remarkable multi-resistance pattern. The number of antibiotic resistances remained very similar year by year with most *SE* strains being multi-resistant to nine antibiotics; a minor representation of low-resistant strains (<5 antibiotics) was found. Therefore, the *SE* population within the INPer hospital was highly stable and led us to question its genetic composition. Specifically, we address the hypothesis that a single or few clones are the basis of the normalization of all the *SE* multi-resistant strains in the children’s health care unit studied.

We analyzed the genomes of 17 selected *SE* strains isolated mostly from neonatal patients. Our findings regarding virulence genetic determinants are concordant with those found in *SE* isolates from hospitals and commensal strains isolated worldwide. The most prominent features of these isolates are their ability to form biofilms, the presence of PMSs, and the multidrug resistance profile displayed (Otto, 2014; Xu et al., 2018). Employing a phylogenetic strategy using the *ccrA, B, C* recombinases, we conclude that most of the INPer *SE* strains carry the SCC*mec* type IV. Surprisingly, eight strains of the *SE* collection analyzed here, harbor neither the *ica* operon nor the IS256, considered pathogenicity markers in *SE* (Kozitskaya et al., 2004; Murugesan et al., 2018). Likely, these are commensal *SE* strains that invaded the patients in the course of their hospital stay even though we cannot discard they use other pathogenicity mechanisms. These strains showed resistance to multiple antibiotics, and three of them contain a composite SCC*mec* cassette formed by type IV and VIII gene elements. We propose a probable structure of the combined SCC*mec* IV and VIII cassette (Fig. S7). It is an unusual combination of SCC elements, but there are reports in the literature of mosaic chromosomal staphylococcal *mec* cassettes (Heusser et al., 2007). However, this SCC*mec* genetic element should be subject to further corroboration. Classification of SCC*mec* is still hard to discern due to the variability and presence of repeated elements, which difficult the correct assembly of the region (International Working Group on the Classification of Staphylococcal Cassette Chromosome E, 2009). The chromosomal cassettes might be hot spots of recombination of heavy metal resistance genes, insertion sequences, and antibiotic resistance genes recruited by HGT (Xue et al., 2017).

The genomic antibiotic resistance spectrum of *SE* strains is very diverse, with some genes present in most strains and others only in few. Examples of diversification of the resistance mechanisms are the presence of membrane efflux pumps (NorAB), which may confer resistance to quinolones as well, and several modifying enzymes (AAC, APH) that inactivate aminoglycosides such as kanamycin. Indeed, the *fosB* gene, which encodes the resistance to fosfomycin was found in 9 out of 17 *SE* strains. In several of these instances, the resistance phenotype coincided with the presence of one or more genes, encoding modifying or degrading enzymes and mutant protein targets for some antibiotics (Table 2).

The genome analysis also indicates some mechanisms for antibiotic resistance, including non-synonymous substitutions in the housekeeping genes *gyrA* and *rpoB*. In the gyrase (*gyrA*) it was found the amino acid S84F change which confers quinolone resistance, whereas, in *rpoB* (*β*-subunit of the RNA polymerase), a double amino acid substitution D471E: I527M, and a single I527M were identified. The double mutant *rpoB* D471E: I527M has been recognized elsewhere as the most common cause of worldwide rifampicin resistance (Lee et al., 2018). Indeed, the presence of this *rpoB* variant reduces the susceptibility to vancomycin and teicoplanin. In this work, the *rpoB* double mutant was detected in S02, S05, and S08 all belonging to ST23 clonal type, while the single mutant I527M was observed in the strains S14, S17, S18, and S19, which are within the ST2 clonal type. Both are the clonal lineages worldwide distributed reported by Leeetal2019. These INPer strains can form biofilms and contain most of the virulence determinants (Table 1). Therefore, they are very adapted *SE* strains and continuously present in the INPer.

In the phylogenetic trees reported, pathogenic *SE* strains are intermingled with commensal *SE* strains with no pathogenic cluster found (Meric et al., 2018; Miragaia, Couto & Lencastre, 2005). Recently, Meric et al. suggested, that pathogenic *SE* subpopulations occur within the commensal *SE* strains, which contain genes and alleles necessary for colonization at distinct infection sites (Meric et al., 2018). These Genome-wide-association (GWAS) studies showed the enrichment of several genes involved with methicillin resistance, biofilm formation, cell toxicity, and inflammatory response in pathogenic *SE* isolates (Meric et al., 2018). Therefore, pathogenic and commensal *SE* strains likely coexist in the same infection site, but current clinical methods of isolation prevent us from distinguishing one from the other. Eight *ica*^−^ out of 17 strains analyzed, could not form biofilms even thought they were recovered from ill patients (Table 1).

Furthermore, here are various footprints of MGEs, including prophages, ISs, and the phage immunity CRISPR-Cas systems in the *SE* genomes that likely contribute to the adaptability by the acquisition of virulence and antibiotic resistance factors. As expected, due to its mobile nature, these elements do not follow a uniform distribution in the phylogeny, indicating frequent genetic exchange in the *SE* population. Together with HGT, homologous recombination may be a factor for genetic diversification of *SE* in hospital settings. In our work, extensive genome analysis of the rates of recombination versus mutation suggests that recombination affects the whole genome and not only a particular class of genes. It has been estimated that recombination could involve 40% of the genome of *SE*, whereas in *S. aureus* recombination comprises the 24% portion (Meric et al., 2015). Although recombination rates depend strongly on the sample of strains used for the analysis, the estimated r/m values reported agrees with other recombination test performed with distinct samples of *SE*, few or whole-genome markers as well as reported r/m numbers in the literature (Meric et al., 2015; Miragaia, Couto & Lencastre, 2005). Therefore we can conclude that the *SE* population despite its whole low level of nucleotide variation (ANIm > 97%) shows cohesive clonal behavior but frequent gene exchange and recombination.

## Conclusions

At local hospital settings, pathogenic, and commensal *SE* strains coexist, but it is hard to discern if they are contaminants, commensal colonizers, or virulent strains (Widerstrom et al., 2016). Indeed, single-colony testing for the identification of *SE* isolates limits to know the extent of multiclonal or multi-species infections (Harris et al., 2016; Van Eldere et al., 2000). In the present study, some analyzed *SE* strains came from nosocomial patients, but lack *ica* genes, a classical virulence marker. However, we cannot exclude that these putative commensal *SE* strains are pathogens in a non-determined manner, or in other conditions. Likely they are part of the intra-hospital non-pathogenic microbiome. These presumed *SE* commensal strains, as well as the biofilm formers considered pathogenic *SE* strains are multi-resistant to antibiotics. The results present here of an 8-year survey, suggest that the multi-resistance to antibiotics might drive adaptation and persistence of specific *SE* clones in hospital settings. HGT and recombination might play a crucial role in the origin of the pathogenic clones, by moving and recombining antibiotic resistance and virulence genes in distinct genomic clonal backgrounds, including non-pathogenic strains. Therefore, the clinical and genetic factors that influence the adaptability stability and change of *SE* community overtime should be addressed in detail in future studies.

##  Supplemental Information

10.7717/peerj.8068/supp-1 Figure S1 Survey of staphylococci at Instituto Nacional de Perinatología, México City Alone eight years A. Species classification and proportion. B. Origin of the isolates and proportion. C. Isolation sites proportion.

10.7717/peerj.8068/supp-2 Figure S2 Frequency cumulation of antibiotic resistant strains in Staphylococcus A. *S. epidermidis*. B. *S. aureus*. The absolute number of antibiotic resistances (x-axis) by the number of strains (y-axis) was counted from 2006 to 2013.

10.7717/peerj.8068/supp-3 Figure S3 Genome identity (ANIm) between pairs of *SE* INPer strains Pairwise whole genome alignments were done with Mummer within the JSspecies program (Richter et al., 2016). The percent of average nucleotide alignment (ANI) was illustrated by a heat-map constructed with ggPlot2 in R (see methods). ANI > 99% are in red color. *S. epidermidis* ATCC 12228 was included for comparison. S10 strain had ANI = 97% respect all the other *SE* strains.

10.7717/peerj.8068/supp-4 Figure S4 Pangenome model of *SE* strains The pangenome model of 29 SE strains was performe with GET_HOMOLOGUES (Contreras-Moreira & Vinuesa, 2013) as described in methods. (A) Pangenome size (number of gene family clusters, Y axis) as a function of the number of *SE* genomes (X axis). (B) Core genome according to the Tettelin equations.

10.7717/peerj.8068/supp-5 Figure S5 Clonal relationships of the STs detected in INPer strains respect to the ST database Alleles for the seven proteins used in the *S. epidermidis* MLST scheme (Thomas et al., 2007) were looked at the *Staphylococcus epidermidis* MLST database (https://pubmlst.org/sepidermidis/; Table 1; see methods) (Feil et al., 2004). The clonal relationships among STs were determined by eBURST (http://eburst.mlst.net). Six out of 8 ST complexes assigned to the *SE* INPer strains are denoted by numbers in violet color.

10.7717/peerj.8068/supp-6 Figure S6 Phylogenetic tree of the SCCmec recombinases A. ccrA. B. ccrB. C. ccrB

10.7717/peerj.8068/supp-7 Figure S7 Proposed structure of the combined cassette SCCmec IV-VIII The segment corresponding to SCCmec cassettes contained within the contig 12 of the S07 strain is annotated according to the best blastN matches against the nr database of the Genebank.

10.7717/peerj.8068/supp-8 Table S1 Reference SCC*mec* and ACME types

10.7717/peerj.8068/supp-9 Table S2 ACME types present in *S. epidermidis*

10.7717/peerj.8068/supp-10 Table S3 Genomes of *S. epidermidis* from the GenBank used in this work

10.7717/peerj.8068/supp-11 Table S4 Genome assembly statistics

10.7717/peerj.8068/supp-12 Table S5 Completeness of *SE* INPer genome collection

10.7717/peerj.8068/supp-13 Table S6 Prophage sequences and their homologs found in *SE* INPer genomes

10.7717/peerj.8068/supp-14 Table S7 CRISPR-Cas elements present in *SE* INPer strains

10.7717/peerj.8068/supp-15 Table S8 Insertion sequences present in *SE* INPer strains

## Additional Information and Declarations

### Data Availability

The following information was supplied regarding data availability: *S. epidermidis* of the INPer collection used in this work are available at GenBank with the following Biosample identifiers: SAMN11086744, SAMN11086745, SAMN11086746, SAMN11086747, SAMN11086748, SAMN11086749, SAMN11086750, SAMN11086751, SAMN11086752, SAMN11086753, SAMN11086754, SAMN11086755, SAMN11086756, SAMN11086757, SAMN11086758, SAMN11086759, SAMN11086760. The accession numbers for the genomes of reference *S. epidermidis* strains are listed in Table S3.
