# Supplementary material for: Genomic diversity of prevalent Staphylococcus epidermidis multidrug-resistant strains isolated from a Children’s Hospital in México City in an eight-years survey
Source: PeerJ. 2019 Nov 20;7:e8068. doi: 10.7717/peerj.8068 (PMC6874853; doi:10.7717/peerj.8068)
Supplement: Figure S1 — A. Species classification and proportion. B. Origin of the isolates and proportion. C. Isolation sites proportion. [file peerj-07-8068-s001.pdf]

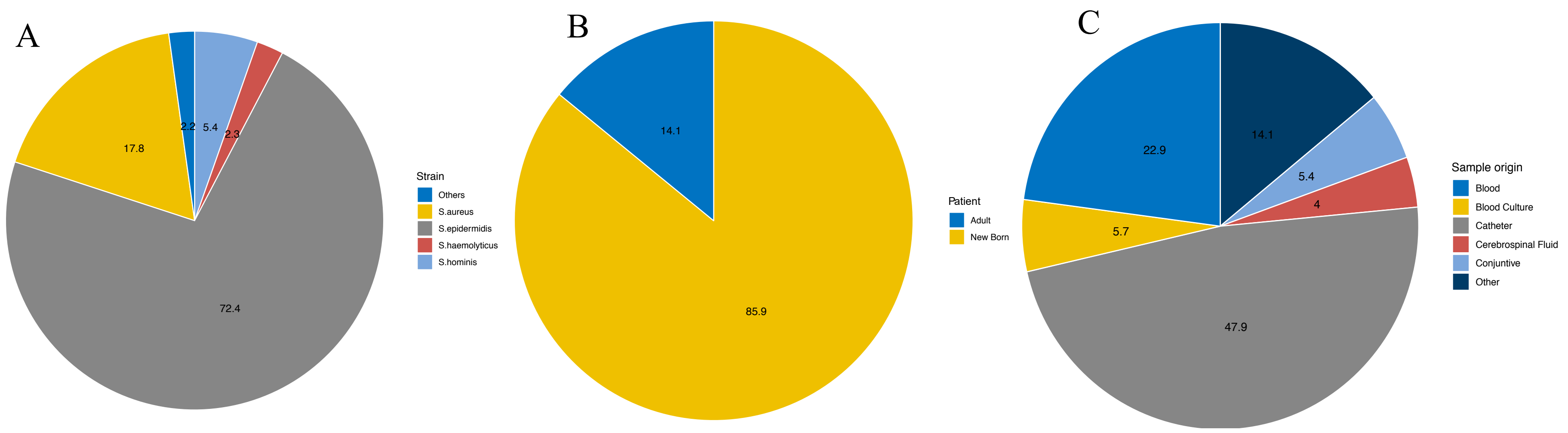
A
2.2
5.4
2.3
17.8
72.4
Strain
Others
S.aureus
S.epidermidis
S.haemolyticus
S.hominis
B
14.1
85.9
Patient
Adult
New Born
C
22.9
14.1
5.4
4
5.7
47.9
Sample origin
Blood
Blood Culture
Catheter
Cerebrospinal Fluid
Conjuntive
Other
