# Supplementary figures and images for: Genomic diversity of prevalent Staphylococcus epidermidis multidrug-resistant strains isolated from a Children’s Hospital in México City in an eight-years survey
Source: PeerJ. 2019 Nov 20;7:e8068. doi: 10.7717/peerj.8068 (PMC6874853; doi:10.7717/peerj.8068)

A

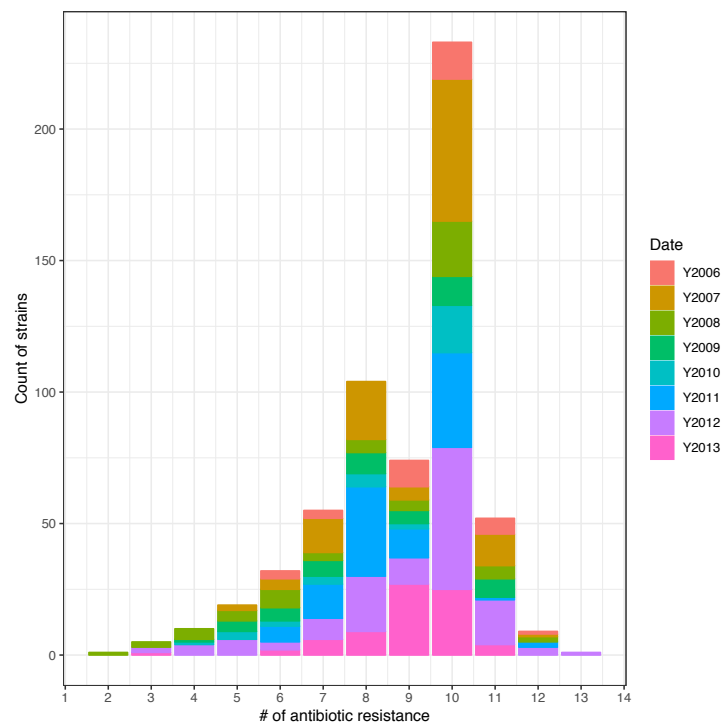

B

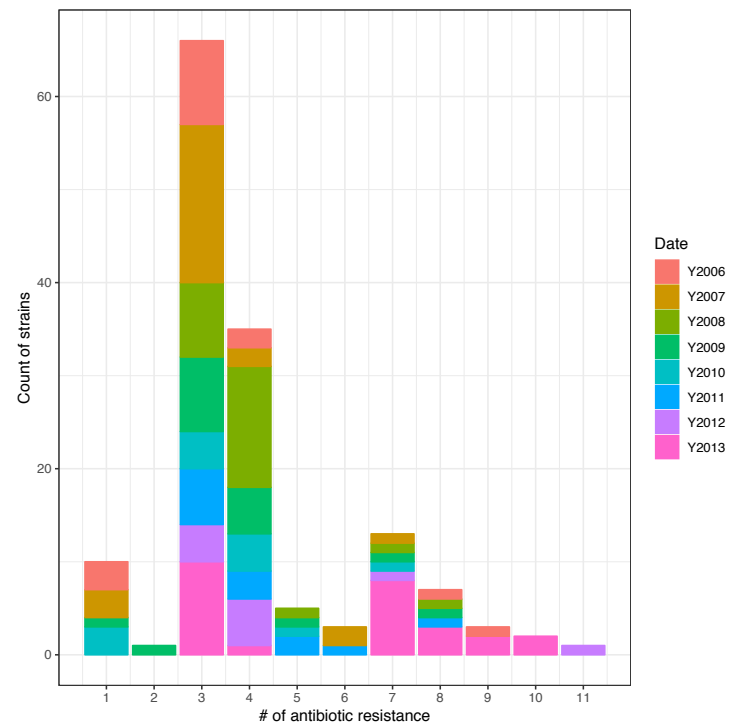

Supplement: Figure S2 — A. S. epidermidis. B. S. aureus. The absolute number of antibiotic resistances (x-axis) by the number of strains (y-axis) was counted from 2006 to 2013. [file peerj-07-8068-s002.pdf]

Figure S1.

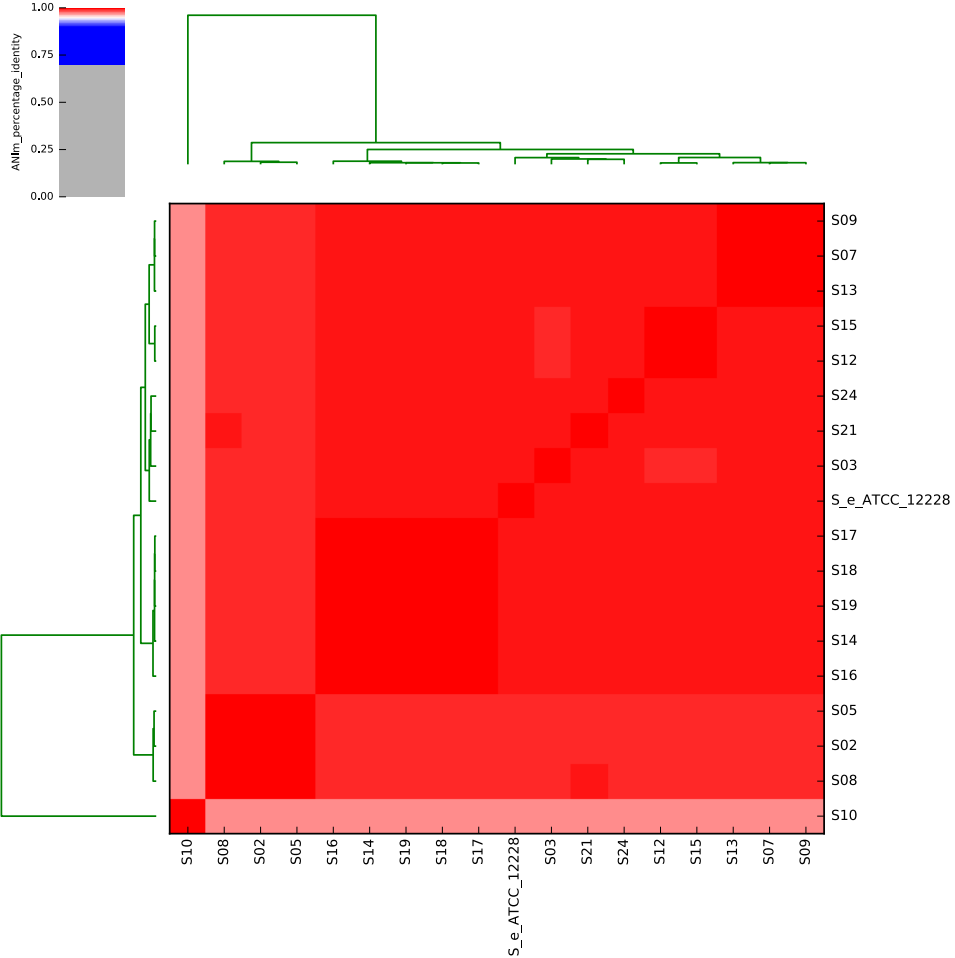

Supplement: Figure S3 — Pairwise whole genome alignments were done with Mummer within the JSspecies program (Richter et al., 2016). The percent of average nucleotide alignment (ANI) was illustrated by a heat-map constructed with ggPlot2 in R (see methods). ANI > 99% are in red color. S. epidermidis ATCC 12228 was included for comparison. S10 strain had ANI = 97% respect all the other SE strains. [file peerj-07-8068-s003.pdf]

Figure S2.

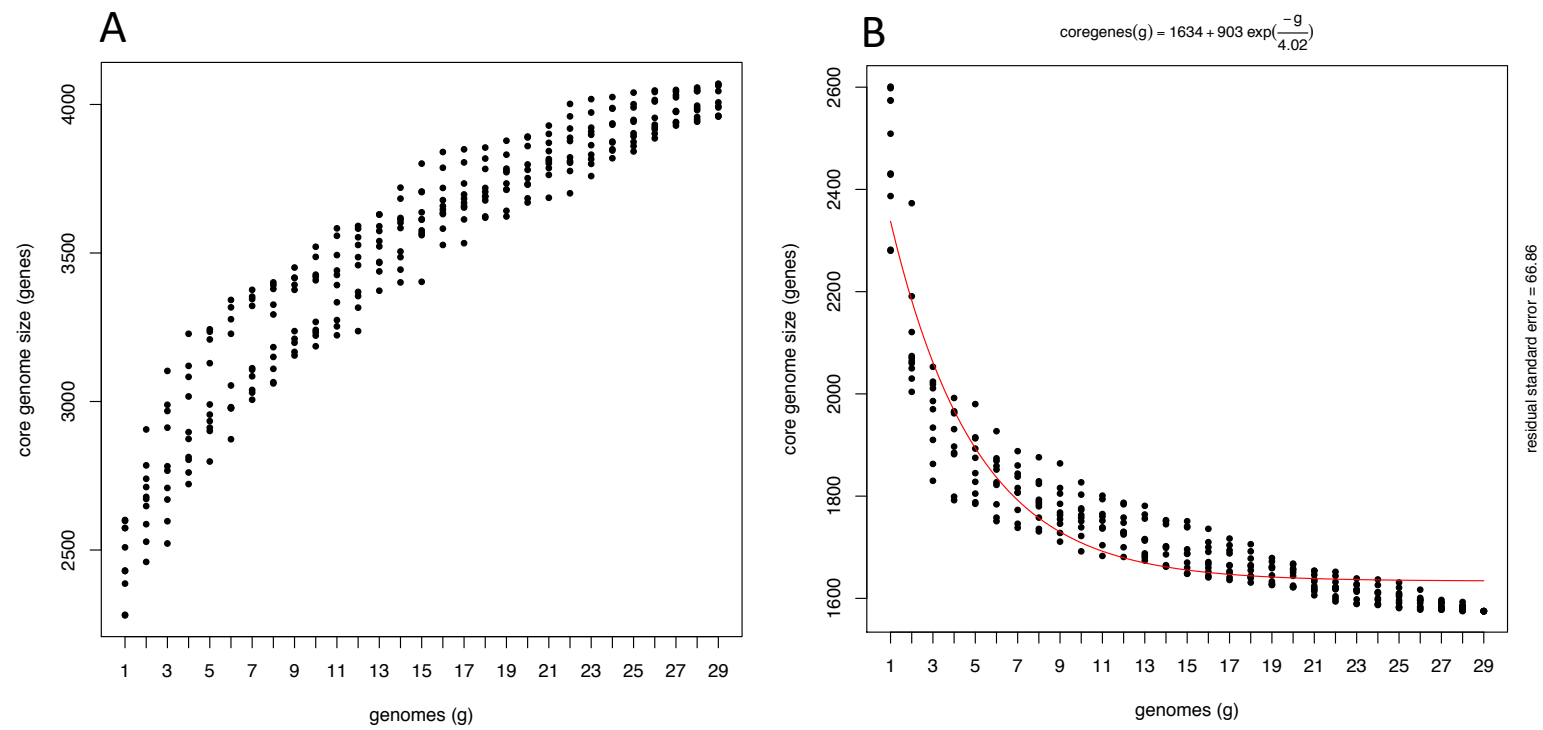

Supplement: Figure S4 — The pangenome model of 29 SE strains was performe with GET_HOMOLOGUES (Contreras-Moreira & Vinuesa, 2013) as described in methods. (A) Pangenome size (number of gene family clusters, Y axis) as a function of the number of SE genomes (X axis). (B) Core genome according to the Tettelin equations. [file peerj-07-8068-s004.pdf]

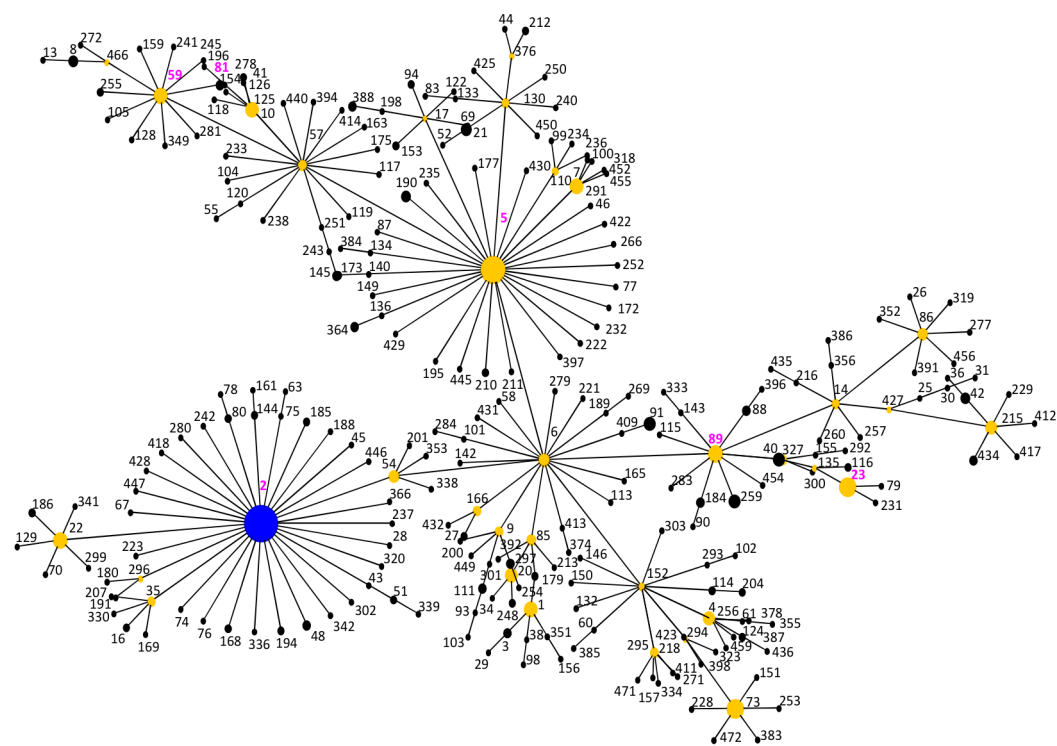

Supplement: Figure S5 — Alleles for the seven proteins used in the S. epidermidis MLST scheme (Thomas et al., 2007) were looked at the Staphylococcus epidermidis MLST database (https://pubmlst.org/sepidermidis/; Table 1; see methods) (Feil et al., 2004). The clonal relationships among STs were determined by eBURST (http://eburst.mlst.net). Six out of 8 ST complexes assigned to the SE INPer strains are denoted by numbers in violet color. [file peerj-07-8068-s005.pdf]

S07 Contig12, 36535 bp, 42 genes

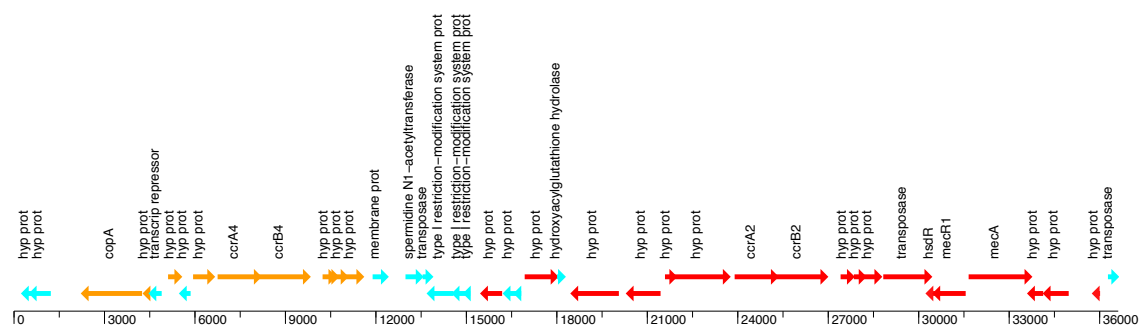

Supplement: Figure S7 — The segment corresponding to SCCmec cassettes contained within the contig 12 of the S07 strain is annotated according to the best blastN matches against the nr database of the Genebank. [file peerj-07-8068-s007.pdf]
