# Supplementary material for: Genomic diversity of prevalent Staphylococcus epidermidis multidrug-resistant strains isolated from a Children’s Hospital in México City in an eight-years survey
Source: PeerJ. 2019 Nov 20;7:e8068. doi: 10.7717/peerj.8068 (PMC6874853; doi:10.7717/peerj.8068)
Supplement: Table S4 [file peerj-07-8068-s011.pdf]

Assembly	Average length read	Insert size deviation	Mean insert lenght	Number of reads	Total number nucleotides	Assembly lenght	Largest contig	Contigs > 300 bp	N50	Number of CDS	CDS lenght
S02	129.46	74.8184	219.763	1306839	169182918	2487658	41096	431	10523	2503	281
S03	131.306	79.8397	227.545	1284904	168715396	2596357	52876	369	15094	2599	279
S05	130.101	75.6184	220.621	1387836	180559144	2497790	126794	316	14378	2463	286
S07	130.659	77.1642	223.431	1373929	179515633	2448467	62390	273	19371	2419	287
S08	133.607	82.608	231.019	919197	122811275	2427136	173888	137	41409	2308	297
S09	129.025	78.2433	223.095	1504056	194060631	2445760	183231	90	72866	2364	292
S10	128.645	78.4613	223.783	1465221	188492958	2518833	177191	115	56447	2464	285
S12	129.78	79.6713	225.552	1480654	192159118	2547392	165170	97	77788	2482	290
S13	127.786	76.9028	220.517	1204712	153945314	2473316	94078	181	30814	2417	289
S14	126.445	70.7468	211.611	1433944	181314690	2531122	43063	523	9299	2593	274
S15	126.85	76.3557	218.561	1501086	190412932	2562288	111209	217	27925	2547	284
S16	126.639	75.3775	217.144	1663934	210719367	2669969	125536	191	34963	2695	279
S17	127.276	73.3061	214.53	1582321	201390878	2558201	63392	331	18380	2569	279
S18	129.074	73.1266	215.52	1577002	203550619	2616140	55887	300	23309	2639	282
S19	130.386	79.8154	226.653	1527471	199160083	2551387	152827	127	61522	2492	290
S21	125.492	75.9589	216.917	1375818	172653544	2436897	208891	77	77274	2320	294
S24	130.153	80.4545	227.012	2030544	264281250	2550997	190522	92	71172	2452	290
