# Supplementary material for: Genomic diversity of prevalent Staphylococcus epidermidis multidrug-resistant strains isolated from a Children’s Hospital in México City in an eight-years survey
Source: PeerJ. 2019 Nov 20;7:e8068. doi: 10.7717/peerj.8068 (PMC6874853; doi:10.7717/peerj.8068)
Supplement: Table S5 [file peerj-07-8068-s012.pdf]

Strain	Scaffolds/Contigs	Genome size	# cds	CDS average lenght
ATCC 12228	7	2564615	2485	286 +- 285
RP62A	2	2643840	2526	287 +- 304
PM221	5	2597508	2508	282 +- 257
SEI	2	2538314	2309	297 +- 300
14.1.R1	4	2625326	2407	286 +- 292
1457	2	2470071	2277	291 +- 206
ATCC 12228(2)	6	2570371	2377	295 +- 297
DAR1907	1	2727549	2502	297 +- 298
FDAARGOS_153	5	2551965	2331	293 +- 222
FDAARGOS_161	3	2515734	2304	299 +- 298
BPH0662	3	2841173	2670	291 +- 298
S02	431	2487658	2503	281 +- 309
S03	369	2596357	2599	279 +- 287
S05	316	2497790	2463	286 +- 315
S07	273	2448467	2419	287 +- 353
S08	137	2427136	2308	297 +- 323
S09	90	2445760	2364	292 +- 355
S10	115	2518833	2464	285 +- 290
S12	97	2547392	2482	290 +- 350
S13	181	2473316	2417	289 +- 353
S14	523	2531122	2593	274 +- 276
S15	217	2562288	2547	284 +- 346
S16	191	2669969	2695	279 +- 277
S17	331	2558201	2569	279 +- 253
S18	300	2616140	2639	282 +- 344
S19	127	2551387	2492	293 +- 269
S21	77	2436897	2320	294 +- 269
S24	92	2550997	2452	290 +- 259

	**Mean GenBank genomes**	**Mean INPer genomes**	**T- statistics**	**P-value**
Genome lenght	2598486	2524689	2.355	0.02605
# cds	2421	2490	-1.618	0.1173
Average CDS lenght	292	286	2.6002	0.01493
