# Supplementary material for: Genomic diversity of prevalent Staphylococcus epidermidis multidrug-resistant strains isolated from a Children’s Hospital in México City in an eight-years survey
Source: PeerJ. 2019 Nov 20;7:e8068. doi: 10.7717/peerj.8068 (PMC6874853; doi:10.7717/peerj.8068)
Supplement: Table S6 [file peerj-07-8068-s013.pdf]

Table S4.

| Strain | # prophage regions | Lenght of the regions | Atachment site | Phage BlastN top hit Name_ID (lenght) | Match lenght (pb) > 95% nucleotide identity |
|---|---|---|---|---|---|
| S02 | 0 | - | - | - | - |
| S03 | 1 | 88.5Kb | Yes | PHAGE_Staphy_SPbeta_like_NC_029119 (127.7 Kb) | 89978 |
| S04 | 0 | - | - | - | - |
| S07 | 1 | 31.8Kb | Yes | PHAGE_Staphy_Ipla5_NC_018281 (43.6 Kb) | 16702 |
| S08 | 0 | - | - | - | - |
| S09 | 0 | - | - | - | - |
| S10 | 0 | - | - | - | - |
| S12 | 1 | 57.3Kb | Yes | PHAGE_Staphy_Ipla7_NC_018284 (42.1 Kb) | 33339 |
| S13 | 0 | - | - | - | - |
| S14 | 1 | 42.6Kb | Yes | PHAGE_Staphy_CNPH82_NC_008722 (43.4 Kb) | 27758 |
| S15 | 1 | 66.6Kb | Yes | PHAGE_Staphy_Ipla7_NC_018284 (42.1 Kb) | 33377 |
| S16 | 1 | 95.5Kb | Yes | PHAGE_Staphy_StB20_NC_019915 (40.9 Kb) | 18716 |
| S16 | 2 | 68.5Kb | Yes | PHAGE_Staphy_SPbeta_like_NC_029119 (127.7 Kb) | 69468 |
| S17 | 1 | 42.6Kb | Yes | PHAGE_Staphy_CNPH82_NC_008722 (43.4 Kb) | 27439 |
| S18 | 1 | 30.2Kb | No | PHAGE_Staphy_CNPH82_NC_008722 (43.4 Kb) | 22885 |
| S19 | 0 | - | - | - | - |
| S21 | 0 | - | - | - | - |
| S24 | 1 | 64.4Kb | Yes | PHAGE_Staphy_Ipla7_NC_018284 (42.1 Kb) | 31691 |
| ATCC 12228 | 0 | - | - | - | - |
| RP62A | 1 | 130.2Kb | Yes | PHAGE_Staphy_SPbeta_like_NC_029119 (127.7 Kb) | 131430 |
| PM221 | 1 | 43Kb | Yes | PHAGE_Staphy_StB20_like_NC_028821 (40.6 Kb) | 23737 |
| SEI | 0 | - | - | - | - |
| 14.1.R1 | 1 | 59.3Kb | Yes | None | - |
| 14.1.R1 | 2 | 44.7Kb | Yes | None | - |
| 1457 | 1 | 40.6Kb | Yes | PHAGE_Staphy_StB20_like_NC_028821 (40.6 Kb) | 35164 |
| ATCC 12228 2 | 0 | - | - | - | - |
| DAR1907 | 1 | 42.8Kb | Yes | PHAGE_Staphy_StB20_NC_019915 (40.9 Kb) | 20898 |
| DAR1907 | 2 | 139.4Kb | Yes | PHAGE_Staphy_SPbeta_like_NC_029119 (127.7 Kb) | 128567 |
| FDAARGOS_153 | 0 | - | - | - | - |
| FDAARGOS_161 | 0 | - | - | - | - |
| CSF41498 | 0 | - | - | - | - |
| BPH0662 | 1 | 43Kb | Yes | PHAGE_Staphy_StB20_NC_019915 (40.9 Kb) | 18538 |
| BPH0662 | 2 | 142.3Kb | Yes | PHAGE_Staphy_SPbeta_like_NC_029119 (127.7 Kb) | 129838 |
| BPH0662 | 3 | 37.5Kb | Yes | None | - |
