# Supplementary material for: Genomic diversity of prevalent Staphylococcus epidermidis multidrug-resistant strains isolated from a Children’s Hospital in México City in an eight-years survey
Source: PeerJ. 2019 Nov 20;7:e8068. doi: 10.7717/peerj.8068 (PMC6874853; doi:10.7717/peerj.8068)
Supplement: Table S7 [file peerj-07-8068-s014.pdf]

Strain	CRISPR array	CAS proteins	Repeat sequence	# repeats	# spacers	Spacer with similarity to a phage
S_epidermis_S02	1	Cas1,Cas2,Csm1,Csm2,Csm3,Csm4,Csm5,Csm6,Cas6	gttctcgtccccttttcttcggggtgggtatcgatc (*)	8	7	spacer #2, Staphylococcus phage PH15
S_epidermis_S03	0	-	-	-	-	-
S_epidermis_S05	1	Cas1,Cas2,Csm1,Csm2,Csm3,Csm4,Csm5,Csm6,Cas6	gttctcgtccccttttcttcggggtgggtatcgatc (*)	6	5	spacer #2, Staphylococcus phage PH15
S_epidermis_S07	0	-	-	-	-	-
S_epidermis_S08	1	Cas1,Cas2,Csm1,Csm2,Csm3,Csm4,Csm5,Csm6,Cas6	tgttctcgtccccttttcttcggggtgggtatcgatc (*)	4	3	None
S_epidermis_S09	1	None	gtaagtggctcttatgcatttttgaaaaaa (+)	3	2	None
S_epidermis_S10	0	-	-	-	-	-
S_epidermis_S12	1	-	tattctcgtcccctgttattcggggtagttatcgatc	10	9	None
S_epidermis_S12	2	Cas1,Cas2,Csm1,Csm2,Csm3,Csm4,Csm5,Csm6,Cas6	gatcgataactaccccgaataacaggggacgagaa (º)	4	3	None
S_epidermis_S13	1	None	gtaagtggctcttatgcatttttgaaaaaa (+)	3	2	None
S_epidermis_S14	0	-	-	-	-	-
S_epidermis_S15	1	Cas1,Cas2,Csm1,Csm2,Csm3,Csm4,Csm5,Csm6,Cas6	gatcgataactaccccgaataacaggggacgagaat (º)	13	12	None
S_epidermis_S16	0	-	-	-	-	-
S_epidermis_S17	0	-	-	-	-	-
S_epidermis_S18	0	-	-	-	-	-
S_epidermis_S19	0	-	-	-	-	-
S_epidermis_S21	1	None	gtaagtggctcttatgcatttttgaaaaaa (+)	3	2	None
S_epidermis_S24	1	Cas1,Cas2,Csm1,Csm2,Csm3,Csm4,Csm5,Csm6,Cas6	gatcgataaccaccccgaagaaaaggggacgagaac	10	9	spacer #6, Staphylococcus phage 6ec
S_epidermis_S24	2	Cas1,Cas2,Csm1,Csm2,Csm3,Csm4,Csm5,Csm6,Cas6	gatcgataactatcccgaagaacaggggatgagaac	5	4	None
