# Supplementary material for: Genomic diversity of prevalent Staphylococcus epidermidis multidrug-resistant strains isolated from a Children’s Hospital in México City in an eight-years survey
Source: PeerJ. 2019 Nov 20;7:e8068. doi: 10.7717/peerj.8068 (PMC6874853; doi:10.7717/peerj.8068)
Supplement: Table S8 [file peerj-07-8068-s015.pdf]

Strain	IS110*	IS1182	IS200/IS605	IS256	IS3	IS6	ISL3	ISNCY	Tn3
S_epidermis_S02	1 (1)	25 (1)	2 (1)	1 (1)	1 (1)	9 (2)		1 (1)	
S_epidermis_S03	2 (1)	26 (2)	2 (1)	1 (1)	10 (2)	10 (3)			
S_epidermis_S05	1 (1)	28 (1)	2 (1)	1 (1)	1 (1)	11 (2)		1 (1)	
S_epidermis_S07	1 (1)	29 (2)	3 (1)		3 (3)	14 (2)			
S_epidermis_S08	1 (1)	27 (1)	2 (2)	1 (1)	1 (1)	7 (2)		1 (1)	
S_epidermis_S09	1 (1)	27 (1)	3 (3)		6 (2)	13 (3)			
S_epidermis_S10	2 (0)	18 (2)	5 (2)		2 (2)	9 (2)	1 (0)		
S_epidermis_S12	1 (1)	27 (1)	2 (1)	1 (1)	7 (3)	5 (2)			
S_epidermis_S13	3 (1)	28 (2)	1 (1)	1 (0)	6 (1)	15 (2)			
S_epidermis_S14	3 (0)	23 (1)	1 (1)	2 (1)	3 (1)	13 (4)			
S_epidermis_S15	2 (1)	30 (1)	5 (4)	1 (1)	7 (3)	6 (2)			
S_epidermis_S16	4 (0)	25 (0)	1 (1)	1 (1)	7 (2)	11 (2)			
S_epidermis_S17	3 (0)	25 (1)	1 (1)	2 (1)	3 (1)	14 (2)			
S_epidermis_S18	3 (0)	26 (1)	1 (1)	2 (1)	3 (1)	17 (3)			
S_epidermis_S19	3 (0)	27 (1)	1 (1)	2 (1)	2 (1)	15 (4)			
S_epidermis_S21	5 (0)	27 (1)	3 (3)		4 (4)	8 (3)			
S_epidermis_S24	3 (0)	28 (1)	2 (2)		5 (3)	14 (2)			
14.1.R1 (GCA_001956655.2)	4 (4)	17 (1)	1 (0)		1 (0)	2 (2)	3 (3)		
1457 (GCA_002085695.1)	9 (9)	11 (7)	3 (3)			6 (6)			
ATCC 12228 (GCA_000007645.1)	15 (14)	38 (2)	4 (3)		4 (1)	3 (3)		1 (1)	
ATCC 12228(2) (GCA_002215535.1)	5 (5)	4 (3)	3 (3)		1 (1)	2 (2)			
BPH0662 (GCA_900086615.1)	6 (6)	10 (3)	2 (2)	2 (2)		10 (9)			2 (2)
DAR1907 (GCA_002850315.1)	9 (9)	21 (0)	2 (2)	2 (2)	4 (4)	10 (6)			
FDAARGOS153 (GCA_002944995.1)	15 (15)		2 (2)			2 (1)		1 (1)	
FDAARGOS161 (GCA_002954055.1)	17 (17)	6 (6)	2 (2)			6 (6)			
PM221 (GCA_000751035.1)	15 (15)	30 (2)	2 (2)			6 (5)			
RP62A (GCA_000011925.1)	13 (11)	13 (0)	2 (2)	5 (5)		9 (7)			
SEI (GCA_000759555.1)	28 (17)	20 (0)	5 (4)		7 (3)	4 (3)			

* Total number of IS elements per family. Parentheses indicate the number of complete ISs.
