# Supplementary material for: Genomic diversity of prevalent Staphylococcus epidermidis multidrug-resistant strains isolated from a Children’s Hospital in México City in an eight-years survey
Source: PeerJ. 2019 Nov 20;7:e8068. doi: 10.7717/peerj.8068 (PMC6874853; doi:10.7717/peerj.8068)
Supplement: Figure S6 — A. ccrA. B. ccrB. C. ccrB [file peerj-07-8068-s006.pdf]

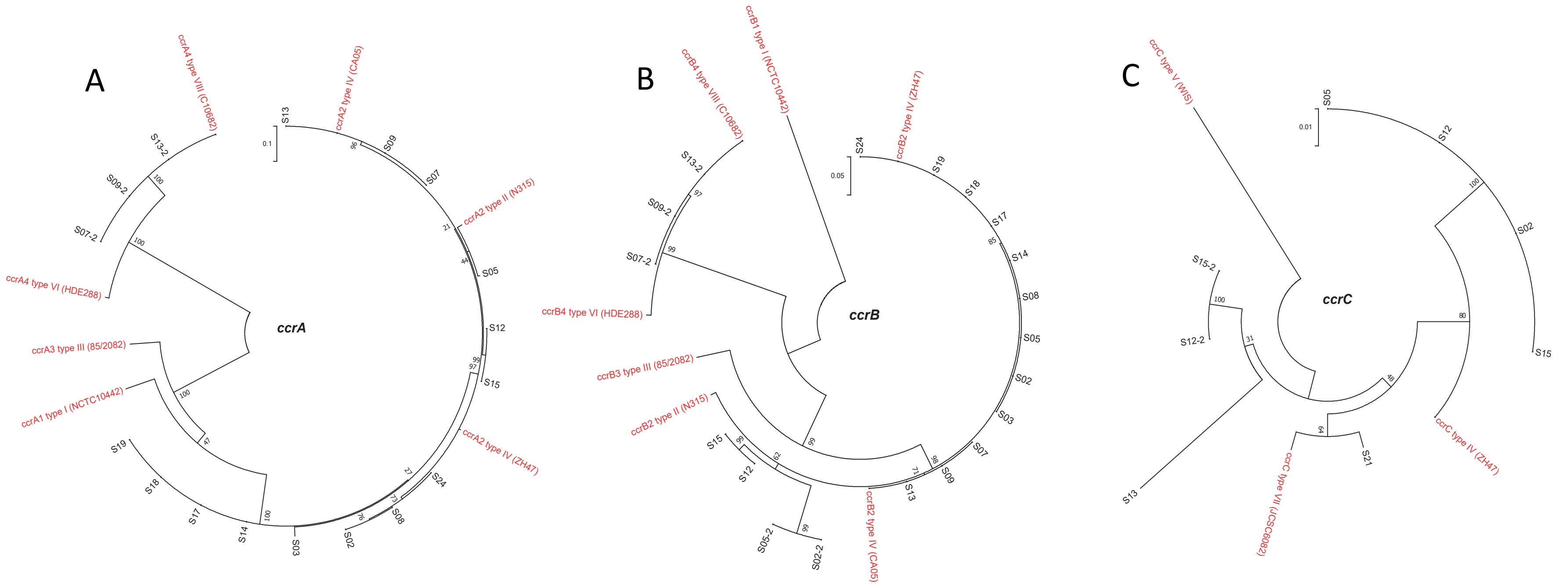
A
ccrA
0.1
S13
ccrA2 type IV (CA05)
S09
S07
ccrA2 type II (N315)
S05
S12
S15
ccrA2 type IV (ZH47)
S24
S08
S02
S03
S14
S17
S18
S19
ccrA1 type I (NCTC10442)
ccrA3 type III (85/2082)
ccrA4 type VI (HDE288)
S07-2
S09-2
S13-2
ccrA4 type VIII (C10682)
B
ccrB
0.05
S24
ccrB2 type IV (ZH47)
S19
S18
S17
S14
S08
S05
S02
S03
S07
S09
S13
ccrB2 type IV (CA05)
S02-2
S05-2
S12
S15
ccrB2 type II (N315)
ccrB3 type III (85/2082)
ccrB4 type VI (HDE288)
S07-2
S09-2
S13-2
ccrB4 type VIII (C10682)
ccrB1 type I (NCTC10442)
C
ccrC
0.01
S05
S12
S02
S15
ccrC type IV (ZH47)
S21
ccrC type VII (JCSC6082)
S13
S12-2
S15-2
ccrC type V (WIS)
